# Circulating Fibroblast Growth Factor 21 and Total Testosterone in Type 2 Diabetes Mellitus Men With Coronary Heart Disease

**DOI:** 10.3389/fendo.2022.912243

**Published:** 2022-07-15

**Authors:** Yufeng Mei, Yongnan Lyu, Zhiming Zhao, Yan Li

**Affiliations:** ^1^ Department of Clinical Laboratory, Renmin Hospital of Wuhan University, Wuhan, China; ^2^ Department of Cardiology, Renmin Hospital of Wuhan University, Wuhan, China; ^3^ Department of Geratology, Renmin Hospital of Wuhan University, Wuhan, China

**Keywords:** FGF21, total testosterone, T2DM, CAD, male patients

## Abstract

**Background:**

Fibroblast growth factor 21 increased in population with type 2 diabetes mellitus (T2DM), while serum total testosterone often decreased in men with T2DM. This study aimed to investigate the relationship between the prevalence of coronary artery disease (CAD) and circulating FGF21 concentrations and serum testosterone in T2DM men.

**Methods:**

490 men with T2DM from January 2021 to December 2021 were recruited from the Renmin Hospital of Wuhan University, and they were divided into CAD group (n=248) and control group (n=242). FGF21 were determined based on ELISA principle and serum total testosterone was measured in a liquid chromatography mass spectrometer LC/MS-8050 (Shimadzu, Japan). Logistic and restricted cubic spline analyses were performed to examine the association between the prevalence of CAD and circulating FGF21 concentrations and serum testosterone in T2DM men. The receiver operating curve (ROC) analysis was used to explore the predictive performance.

**Results:**

Circulating FGF21 levels were higher in T2DM men with CAD compared with those without CAD [214.63 (121.82, 348.64) pg/ml vs 166.55 (94.81,254.48) pg/ml, *p*<0.001], while serum total testosterone was lower [3.08 ± 0.07 ng/ml vs 3.76 ± 0.09 ng/ml, p<0.001]. The fully adjusted odds ratio (OR) and 95% confidence intervals (95%CI) was 2.956(1.409,6.201) for those in quartile 4 of FGF21 versus quartile 1 and the fully adjusted OR (95%CI) was 0.346(0.174,0.686) for those in quartile 4 of testosterone versus quartile 1. The receiver operating curve (ROC) analysis showed that the area under the curve (AUC) of combination of FGF21 and testosterone for predicting the occurrence of CAD in men with T2DM was 0.702 (95% CI: 0.667-0.741).

**Conclusion:**

Circulating FGF21 levels were positively associated with CAD in men with T2DM, whereas serum total testosterone levels showed an inverse correlation with CAD in diabetic men.

## Introduction

With improvements of living standards and dietary diversification, metabolic diseases, especially the type 2 diabetes mellitus (T2DM), have seriously threatened the global economy and social public health. Further studies have demonstrated that gender, mainly androgen, affected differently on T2DM, manifesting as low serum testosterone levels in male T2DM patients (1–3). Interventional researches have also pointed that testosterone replacement therapy (TRT), to varying degrees, improves glucose metabolism disorders in T2DM men (4). In addition, due to the close connection of glucose and lipid metabolism, coronary artery disease (CAD) has become a common and serious complication of T2DM. Epidemiological data suggest that, in men, testosterone plays a role similar to that in T2DM in the development of CAD, and TRT helps reduce future adverse cardiovascular events (5–7). Therefore, testosterone may serve as an effective biological indicator in the prevention and management of CAD in T2DM men.

FGF21 is a metabolic molecule closely related to T2DM. In glucose metabolism, it has a beneficial effect on T2DM patients by participating in glucose homeostasis as a signal molecule and regulating the sensitivity of pancreatic β cells. At the same time, in lipid metabolism, it can also reverse hyperlipidemia by Akt, AMPK and other signal transduction pathways, which has been positively confirmed in animal experiments (8–10). Furthermore, clinical studies revealed a potential association of high circulating levels of FGF21 in serum with atherosclerosis as well as CAD, independent of established cardiovascular risk factors (11, 12).

However, there is currently a lack of evidence that serum testosterone concentrations as well as FGF21 levels are associated with CAD in men with T2DM. Therefore, this study analyzed men with T2DM to explore the relationship between circulating FGF21 and testosterone and CAD.

## Methods

### Participants

All participants were rolled from the Department of Endocrinology and Cardiovascular Medicine, People’s Hospital of Wuhan University. The exclusion criteria follows: (1) severe infection such as sepsis, (2) cancer or undergoing radiotherapy and chemotherapy, (3) congenital metabolic disorders, and autoimmune diseases, (4) uremia or other severe renal disease or history of acute kidney injury after percutaneous coronary intervention, (5) recent history of surgery or brain trauma, severe physical and cerebral organic disease, (6) receiving liver transplantation or other severe liver disease, (7) recent use of hormones drugs, (8)incomplete basic information. After exclusion, totally 490 patients diagnosed with T2DM from January 2021 to December 2021 were included in the study and they were divided into controls group (n=242) and CAD group (n=248).

### Study Definition of Clinical Variables

The definition of CAD was consistent with the World Health Organization/International Society and Federation of Cardiology Working Group (the coronary stenosis ≥ 50% in three major coronary arteries) (13), with diagnosis adjudicated and reviewed by two physicians and differences resolved by the third. The diagnosis of T2DM conformed to the American Diabetes Association (ADA) criteria (FPG ≥ 7.0mmol/L, 2hPG ≥ 11.11mmol/L, HbA1c ≥ 6.5% and random plasma glucose ≥ 11.1mmol/L) (14). Drinkers or smokers referred to those who did not stop their drinking or smoking from the past to the present. BMI was calculated as weight (kg) divided by height (meters) squared. Hypertension was defined as systolic/diastolic blood pressure ≥140/90 mmHg after patient had been sitting for at least 10 minutes (15).

### Blood Sampling and Laboratory Analysis

All subjects after admission fasted for at least 8 hours and underwent venipuncture at 8-11am the following morning. Serum was collected by centrifugation for 5 min after incubation of blood samples for 15 min at room temperature and then stored at -80°C until measurement. 20ul serum was added to 220ul mixed system consisting of acetonitrile and internal standard. After mixing, vortexing and centrifugation, the supernatant was collected and tested for testosterone in a liquid chromatography mass spectrometer LC/MS-8050 (Shimadzu, Japan). FGF21 was measured using a commercial enzyme-linked immunosorbent (ELISA) kit purchased from R&D system (Quantikine, R&D Systems, USA) with a linear detection range of 31.3 pg/ml-2000 pg/ml, intra- and inter-assay coefficients of variation 2.9-3.9% and 5.2-10.9% respectively. The quantitative principle of this kit was the double antibody sandwich method and the color development system included streptavidin-HRP, hydrogen peroxide and tetramethylbenzidine. All reagents were stored at 2-8°C before the measurement.

The determination principle of HbA1c was ion exchange high performance liquid chromatography, and total cholesterol (TC), triglycerides (TG), free fatty acids (FFA), glucose (Glu), alanine aminotransferase (ALT), aspartate aminotransferase (AST), high density lipoprotein cholesterol (HDL-C), low density lipoprotein cholesterol (LDL-C), Urea, creatinine (Cr) and high-sensitivity C-reactive protein (hs-CRP) were quantified on a Siemens automatic biochemical analyzer ADVIA 2400 (Erlangen, Germany). We calculated eGFR using the CKD-EPI Cr formula recommended by the National Kidney Foundation (NKF) and the American Society of Nephrology (ASN) Task Force (16).

### Statistical Analysis

Analysis were performed with R version 3.6.3 (www.r-project.org), SPSS software version 23.0 (IBM, Armonl, NY, USA) and GraphPad Prism 7.0 (GraphPad Software, La Jolla, CA, USA). According to the Kolmogorov–Smirnov test, BMI and total testosterone were normally distributed and these two variables were therefore presented as mean ± standard deviation, then analyzed with Student t-test. Other clinical and biochemical parameters did not obey the normal distribution and they were expressed as interquartile range, analyzed with the Mann-Whitney test. Hypertension, anti-diabetic drugs, statin, smokers, and drinkers were showed as percentages, and the chi-square test was therefore performed for comparison. Univariate and multivariate logistic regression were conducted to investigate the association between the prevalence of CAD and circulating FGF21 concentrations and serum testosterone in T2DM men. To further explore the relationship of FGF21 and testosterone with the prevalence of CAD in T2DM, we conducted a restricted cubic spline analysis in a fully adjusted model. Finally, the receiver operating curve (ROC) analysis was used to assess the predictive performance of FGF21 and testosterone for CAD.

## Results

### Sample Characteristics

**Table 1** summarized the clinical and biochemical characteristics of 490 T2DM participants. In terms of clinical data, diabetic men with CAD had a higher onset age, diabetes duration and BMI than those with T2DM only (*p*<0.01), but there was no difference in clinical parameters such as smoking, alcohol drinking, anti-diabetic drugs, statin and hypertension. As regard to biochemical parameters, there was no difference in FPG and HbA1c between the two groups and diabetic men with CAD had higher levels of AST, FFA, Urea, Cr, hsCRP and FGF21 (*p* all <0.001), while HDL-C, eGFR and testosterone levels were lower (*p* all <0.01), unmasking existence of inflammation, unfavorable lipid metabolism, and relatively poor renal function in diabetic men with CAD.

**Table 1:** Clinical and Biochemistry characteristics of all subjects.

Characteristics	T2DM without CAD (n = 242)	T2DM with CAD (n = 248)	*p value*
Age (years)	53 (42,59)	59 (52.67)	<0.001
BMI (kg/m^2^)	24.39 ± 0.11	24.89 ± 0.14	0.008
Diabetic durations (years)	1 (0,4)	3 (0,6)	<0.001
Hypertension (%)	30.58	33.06	0.562
Smoking (%)	16.94	18.95	0.638
Alcohol consumption (%)	13.64	13.71	0.981
Anti-diabetic drugs (%)	38.02	34.68	0.442
Statin (%)	25.62	25.81	0.962
HbA1c (%)	8.6 (6.7,10.4)	8.8 (8.4,10.0)	0.323
FPG (mmol/L)	9.85 (7.27,12.47)	9.04 (7.47,11.32)	0.271
ALT (U/L)	19 (14,28)	21 (15,29)	0.076
AST (U/L)	17 (14,22)	21 (16,28)	<0.001
FFA (mmol/L)	0.30 (0.20,0.45)	0.55 (0.33,0.91)	<0.001
TC (mmol/L)	4.51 (3.83,5.12)	4.51 (3.79,5.34)	0.550
TG (mmol/L)	1.53 (1.05,2.65)	1.56 (1.07,2.32)	0.941
HDL-C (mmol/L)	0.91 (0.80,1.10)	0.87 (0.75,1.02)	0.007
LDL-C (mmol/L)	2.35 (1.77,3.03)	2.40 (1.69,3.10)	0.808
Urea (mmol/L)	5.45 (4.56,6.29)	5.89 (4.76,7.03)	<0.001
Cr (μmol/L)	64 (57,72)	68 (60,78)	<0.001
eGFR (ml/min/1.73m^2^)	106.90 (98.32,115.99)	97.63 (90,57,106.84)	<0.001
hsCRP (mg/dl)	0.88 (0.34,2.86)	1.69 (0.63,4.22)	<0.001
Testosterone (ng/ml)	3.76 ± 0.09	3.08 ± 0.07	<0.001
FGF21 (pg/ml)	166.55 (94.81,254.48)	214.63 (121.82,348.64)	<0.001

Data were presented with numbers (percentages) for categorical variables and median (interquartile range) for continuous variables.

BMI, body mass index; HbA1c, glycated hemoglobin A1c; FPG, fasting plasma glucose; ALT, alanine aminotransferase; AST, aspartate aminotransferase; FFA, free fatty acid; TC, total cholesterol; TG, triglyceride; HDL-c, high-density lipoprotein cholesterol; LDL-c, low-density lipoprotein cholesterol; Cr, creatinine; eGFR, estimated glomerular filtration rate (mL/min/1.73 m2); hsCRP, high-sensitivity C-reactive protein; FGF21, fibroblast growth factor 21.

### Association Between the Prevalence of CAD and Circulating FGF21 Concentrations and Serum Testosterone in T2DM Men

**Table 2** depicted the odds ratio (OR) and 95% confidence intervals(95%CI) for the prevalence of CAD for circulating FGF21 concentrations, and analysis was based on FGF21 quartiles, taking participants in the first FGF21 quartiles as the reference. Likewise, **Table 3** presented the OR and 95% CI for the prevalence of CAD for serum total testosterone. In the crude model with no adjustments, FGF21 levels were positively associated with the prevalence of CAD in men with diabetes, whereas serum total testosterone concentrations showed the opposite results contrary to FGF21. After adjusting for BMI, diabetic duration, age, hypertension, smoking, anti-diabetic drugs, statin and drinking status in Model 1, the characteristics were consistent with those in crude model. After additional adjustment for FPG, FFA, HbA1c, TC, HDL-C, ALT, AST, TG, LDL-C, Cr, eGFR, Urea, and hsCRP in model 2, this correlation changed slightly and remained statistically significant. The OR (95%CI) of CAD in model 2 was 2.956 (1.409,6.201) in quartile 4 (the highest) versus quartile 1 (the lowest) of circulating FGF21 concentrations. The fully adjusted OR (95%CI) in Model 2 was 0.346 (0.174,0.686) for those in quartile 4 of serum testosterone (the highest) versus quartile 1 (the lowest).

**Table 2:** Association of CAD with serum FGF21 in T2DM men.

FGF21 quartiles	n	Concentration range	OR (95%CI)		
			Crude	Model 1	Model 2
Quartile 1 (low)	122	<107.08	reference	reference	reference
Quartile 2	123	107.08-190.84	2.247 (1.334,3,784)	2.580 (1.447,4.600)	1.627 (0.807,3.280)
Quartile 3	122	190.84-303.81	2.645 (1.569,4.459)	3.287 (1.838,5.889)	2.122 (1.038,4.339)
Quartile 4 (high)	123	>303.81	3.527 (2.078,5.987)	4.518 (2.499,8.168)	2.956 (1.409,6.201)
*p* value			<0.001	<0.001	0.004

Crude: no adjustment.

Model 1: adjusted for age, diabetic durations, BMI, alcohol drinking, smoking, hypertension, anti-diabetic drugs and statin.

Model 2: adjusted for the same variables as Model 1 as well as HbA1c, FPG, ALT, AST, Urea, Cr, eGFR, FFA, TC, TG, HDL-C, LDL-C and hsCRP.

**Table 3:** Association of CAD with serum total testosterone in T2DM men.

Testosterone quartiles	n	Concentration range	OR (95%CI)		
			Crude	Model 1	Model 2
Quartile 1 (low)	122	<2.429	reference	reference	reference
Quartile 2	123	2.429-3.398	0.285 (0.168,0,483)	0.243 (0.135,0.436)	0.280 (0.138,0.570)
Quartile 3	122	3.398-4.306	0.382 (0.227,0.642)	0.271 (0.155,0.488)	0.303 (0.149,0.617)
Quartile 4 (high)	123	>4.306	0.480 (0.286,0.805)	0.364 (0.205,0.647)	0.346 (0.174,0.686)
*p* value			<0.001	<0.001	0.001

Crude: no adjustment.

Model 1: adjusted for age, diabetic durations, BMI, alcohol drinking, smoking, hypertension, anti-diabetic drugs and statin.

Model 2: adjusted for the same variables as Model 1 as well as HbA1c, FPG, ALT, AST, Urea, Cr, eGFR, FFA, TC, TG, HDL-C, LDL-C and hsCRP.

### Restricted Cubic Spline Analysis

**Figure 1:**
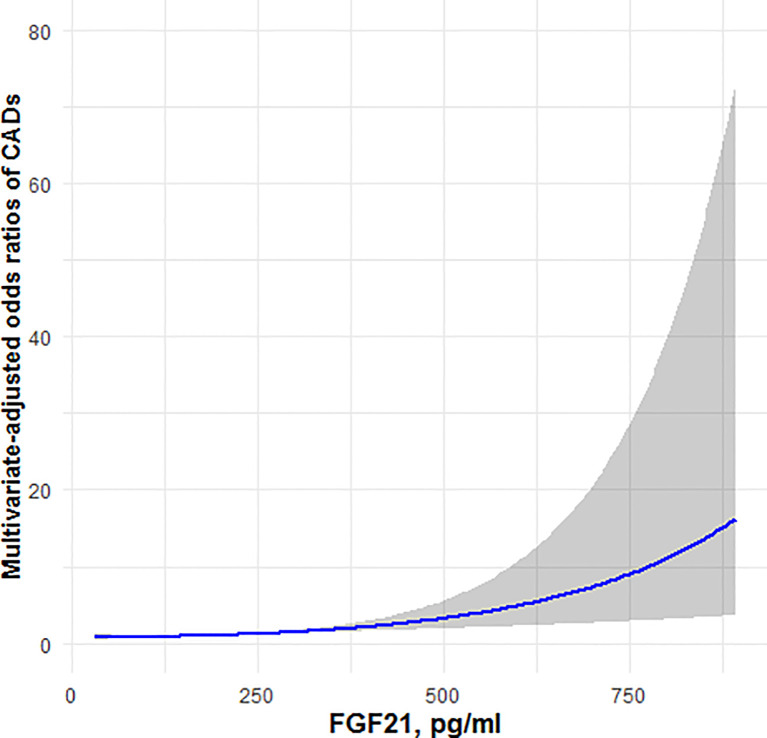
Restricted cubic spline model of the odds ratios of CAD with circulating FGF21. The gray areas represent the 95% confidence intervals.

**Figure 2:**
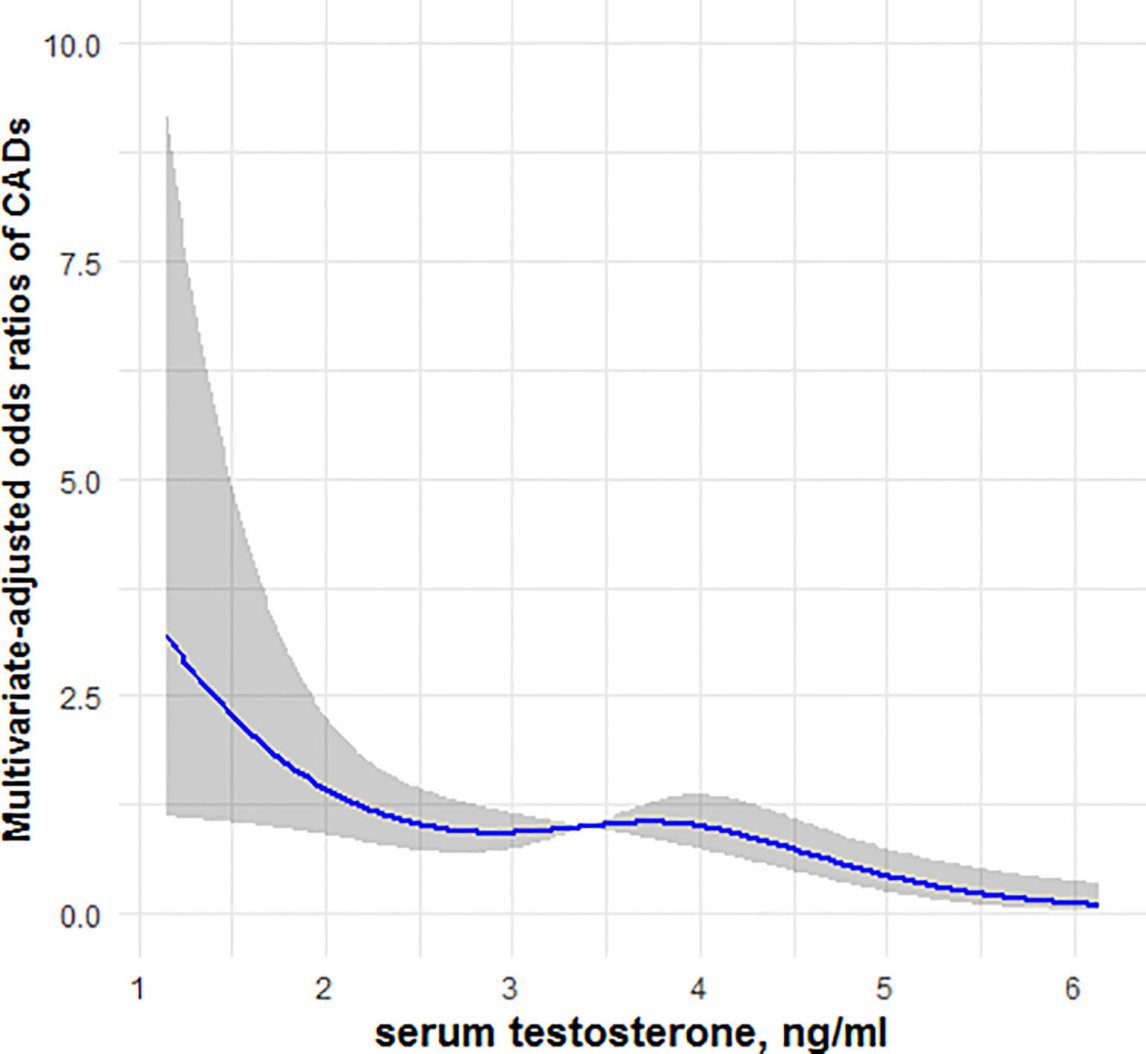
Restricted cubic spline model of the odds ratios of CAD with serum testosterone. The gray areas represent the 95% confidence intervals.

**Figures 1**, **2** showed, respectively, the association of circulating FGF21 levels and serum testosterone concentrations with CAD using restricted cubic spline analysis in male patients with T2DM. The results showed that circulating FGF21 levels were positively associated with the prevalence of CAD in men with T2DM, after adjusting for all potential confounders in this study, whereas serum testosterone concentrations were negatively associated with the prevalence of CAD in men with T2DM, indicating an L-shaped association.

### ROC Analysis

**Figure 3:**
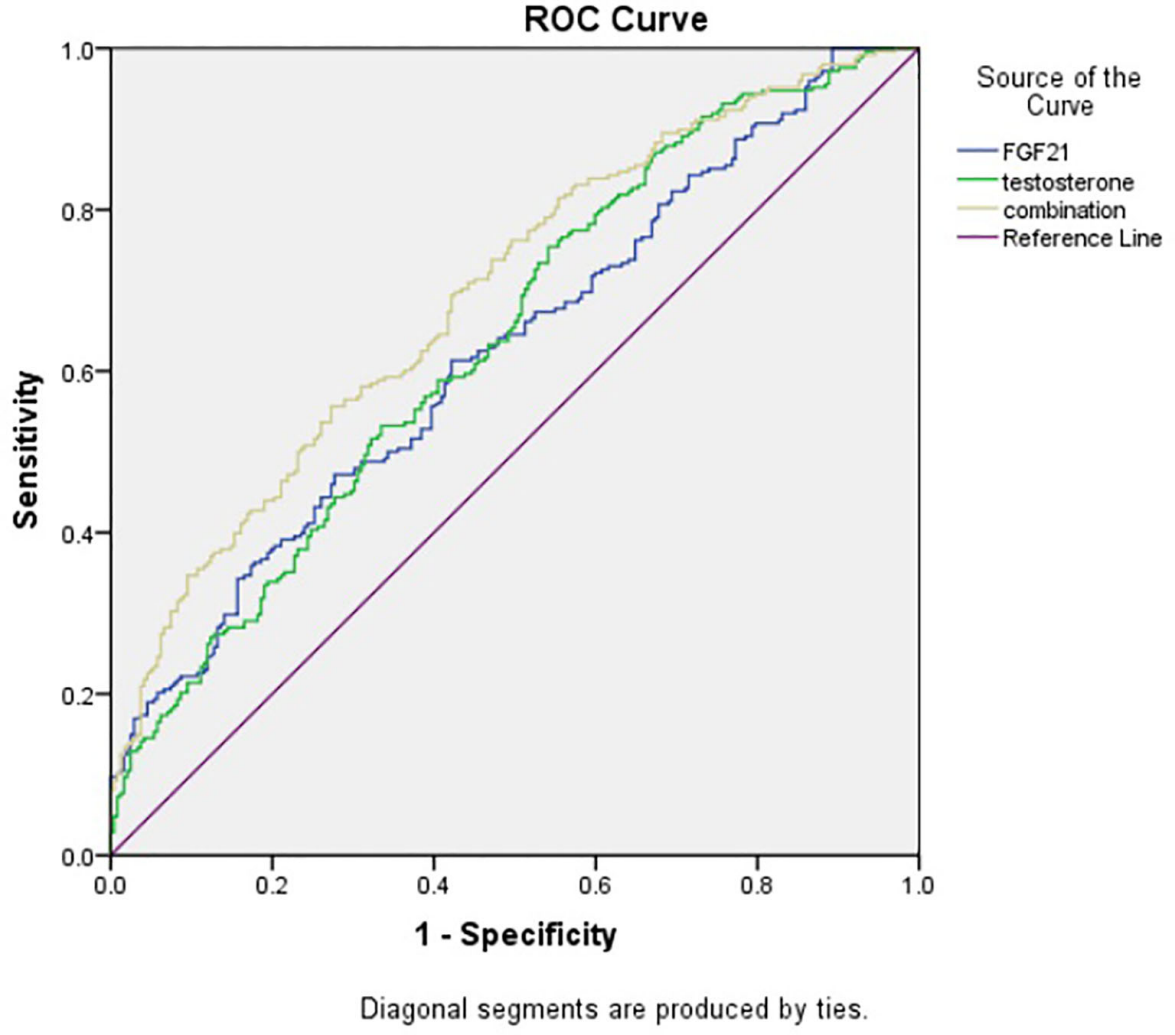
ROC curve of serum FGF21 and testosterone in predicting the occurrence of CAD in T2DM men.

As shown in **Figure 3**, the ROC curve depicted that the area under the curve (AUC) of serum FGF21 in predicting the occurrence of CAD in T2DM men was 0.625 (95% CI: 0.576-0.674), the sensitivity was 0.472, the specificity was 0.723, and the Youden index was 0.195. The AUC of testosterone for predicting CAD in men with T2DM was 0.640 (95% CI: 0.591-0.688), the sensitivity was 0.754, the specificity was 0.459, and the Youden index was 0.213. In addition, the AUC of combination of FGF21 and testosterone for predicting the occurrence of CAD in men with T2DM was 0.702 (95% CI: 0.667-0.741), with sensitivity 0.552, specificity 0.727, and Youden index 0.280.

## Discussion

In this current study, baseline data from 490 male patients diagnosed with T2DM were analyzed to investigate the relationship between CAD and circulating FGF21 levels or testosterone concentrations. We found that, in men with T2DM, serum FGF21 concentrations were positively associated with the prevalence of CAD, whereas total testosterone levels in circulation were negatively associated with the prevalence of CAD, and these associations remained statistically significant after controlling for potential confounders. This is the first study to investigate the relationship between the prevalence of CAD in men with T2DM and circulating FGF21 concentrations and serum total testosterone levels. This study is also the first to find that the combination of FGF21 and testosterone may be the potential predictive marker in the occurrence of CAD in men with T2DM.

FGF21 was defined in 2004 as a metabolic molecule, and cloned as a differentiated member of the FGF19 subfamily (17). Human’s FGF21 gene was located on chromosome 7 and contained 3 exons and the expressing product of this gene participated in various cellular activities through FGF21 receptor 1 (FGFR1) and β-klotho heteroreceptor complex, such as apoptosis, autophagy, inflammation, lipid catabolism, angiogenesis regulation and glucose homeostasis (18–20). FGF21 expressed in multiple tissues, for instance the liver, pancreas, adipose, cardiac and skeletal muscle (21). Given the pleiotropic effects of FGF21, especially the close relation to lipid and glucose metabolism, detecting circulating FGF21 was the reflection of poor lipid metabolism and glucose metabolism in T2DM patients, which may explain the potential link between FGF21 and CAD, because lipid disorder was a risky factor for CAD in T2DM (22). Consistent with the previous reports, FFA in this research was elevated in CAD while the beneficial lipid HDL-C was decreased.

Several studies have revealed the intrinsic link between FGF21 and CAD. Zhang Y et al. analyzed a large population and discovered that high levels of circulating FGF21 were strongly associated with increased risk of CAD (23). A cross-sectional study in a Chinese population unmasked that serum FGF21 levels in CAD patients increased with the severity of metabolic disorders, independent of age, gender and BMI (24). In accordance with previous findings, results in our research supported that high concentrations of FGF21 in men with T2DM were associated with CAD, an association that remained statistically significant after controlling for multiple confounding variables, thus speculation came that FGF21 may be involved in pathogenesis of CAD in T2DM patients.

Despite FGF21 was closely related to T2DM and CAD, the specific mechanism by which FGF21 participated in the occurrence of CAD has not been elucidated, and study mainly referred to atherosclerosis. Dai Q et al. revealed that FGF21 increased the content of nicotinamide adenine dinucleotide (NAD+) through an AMPK-dependent pathway and improved hyperglycemia-related ischemic angiogenesis and endothelial progenitor cell (EPC) function, which was critical for investigating T2DM complications, especially CAD (25). *In vitro*, FGF21 increased plaque RACK1 and autophagy-related proteins, such as beclin-1 and LC3, thereby inducing autophagy-mediated cholesterol efflux and inhibiting foam cell development, while foam cells gathering in vascular wall has been proved an important link in the pathogenesis of CAD (19). Another animal experiment showed that FGF21 reduced ROS production and alleviated atherosclerosis possibly by reducing NLRP3 inflammasome-mediated pyroptosis of vascular endothelial cell, maintaining normal mitochondrial dynamics, and inhibiting VEC endoplasmic reticulum stress (26). In addition, FGF21 might inhibit hepatic expression of sterol regulatory element-binding protein 2 and increase adiponectin levels, then effectively improved atherosclerosis and prevented obesity-related metabolic heart disease (27, 28). These literatures not only suggested that high circulating FGF21 were adaptive increase in response to metabolic disturbances, but also implied that monitoring serum FGF21 levels may serve as an effective target for managing CAD in men with T2DM.

The relationship between testosterone and CAD in recent years has been gradually recognized. As an important endogenous vasodilator in men, testosterone regulates coronary arteries through genomic and non-genomic pathways. The former mainly binds to androgen receptor (AR) across the plasma membrane, upregulating subsequent transcription and protein expression levels, and the latter refers to protein/receptor/ion channel interactions at the plasma membrane (29). Previous findings have demonstrated that serum testosterone levels decreased in men with T2DM or CAD, but it is unclear how testosterone changes in men with T2DM and CAD versus men with T2DM alone (2, 3, 6, 7). It’s noted for the first time in our research that T2DM men with CAD had lower serum testosterone levels than those with T2DM merely, further supporting the potential importance of testosterone in diabetes-related CAD.

Testosterone is closely linked to CAD and glucose metabolism. There has been evidence that testosterone decreasing in men with T2DM might be involved in CAD by accelerating ROS production, promoting mitochondrial dysfunction and enhancing inflammatory interactions between vascular endothelium and leukocytes (30). Hotta Y et al. pointed out that testosterone deficiency affected the endothelial repair system, possibly *via* increasing asymmetric dimethylarginine, regulating nitric oxide synthase expression, and proliferation and migration of EPCs (31). Furthermore, Troncoso MF et al. used testosterone to stimulate cardiomyocytes *in vitro* and determined that testosterone accelerated glucose consumption by activating AMPK and AR signaling pathways, suggesting that the response to testosterone deficiency might be a key factor promoting CAD occurrence in men with T2DM (32). Indeed, aromatase-mediated accelerated aromatization of testosterone to estradiol might also have an impact on the development of CAD (33, 34), since testosterone supplementation greatly improved insulin resistance and reduced the risk of CAD (35, 36). Therefore, monitoring changeable serum testosterone level in T2DM men possibly provides a personalized perspective on CAD.

Previous studies ignored the gender differences in T2DM and CAD. This study was based on men with T2DM and not only found that an elevated level of FGF21 was a potential risk factor for CAD in men with T2DM, but more importantly, unmasked for the first time that a persistently declined level of testosterone in T2DM men might be a potential predictor of CAD. Hence, monitoring these two serum indicators might be of great clinical significance for the identification and prevention of CAD. Nevertheless, this research has several limitations. First, it has a small sample, which means it needs to be corroborated in a larger population. Another limitation was the lack of measurements on normal population. In addition, there may be some undetected confounding factors affecting FGF21 and testosterone, such as NAFLD (20), which possibly amplify bias. Finally, the applicability of findings in this study to populations of other races and regions is unknown.

## Conclusion

In short, high circulating FGF21 levels and low serum total testosterone may be associated with CAD in men with T2DM. Monitoring serum FGF21 and testosterone may be an effective means to prevent and manage CAD in men with T2DM.

## Data Availability Statement

The datasets presented in this article are not readily available due to privacy or ethical restrictions. Requests to access the datasets should be directed to the corresponding author.

## Ethics Statement

The studies involving human participants were reviewed and approved by the Medical Ethics Review Committee of Renmin Hospital of Wuhan University, China. The patients/participants provided their written informed consent to participate in this study.

## Author Contributions

YM: Data collection and curation, statistical analysis, writing – original draft, and writing – review and editing. YLyu: Statistical analysis and supervision. ZZ: Data checking, statistical analysis, validation and supervision. YLi: Funding acquisition, supervision, and validation. All authors contributed to the article and approved the submitted version.

## Funding

This research was supported by the National Natural Science Foundation of China (Grant Numbers: 81772265).

## Conflict of Interest

The authors declare that the research was conducted in the absence of any commercial or financial relationships that could be construed as a potential conflict of interest.

## Publisher’s Note

All claims expressed in this article are solely those of the authors and do not necessarily represent those of their affiliated organizations, or those of the publisher, the editors and the reviewers. Any product that may be evaluated in this article, or claim that may be made by its manufacturer, is not guaranteed or endorsed by the publisher.

## References

[1] Liu X Jiang J Liu X Luo Z Wang Y Dong X. Association of Serum Testosterone With Different Classes of Glucose Metabolism and the Mediation Effect of Obesity: The Henan Rural Cohort Study. Diabetes Metab Res Rev (2019) 35(5):e3133. doi: 10.1002/dmrr.3133 30715782

[2] Ho CH Jaw FS Wu CC Chen KC Wang CY Hsieh JT. The Prevalence and the Risk Factors of Testosterone Deficiency in Newly Diagnosed and Previously Known Type 2 Diabetic Men. J Sex Med (2015) 12(2):389–97. doi: 10.1111/jsm.12777 25441980

[3] Zhang J Li X Cai Z Li H Yang B. Association Between Testosterone With Type 2 Diabetes in Adult Males, A Meta-Analysis and Trial Sequential Analysis. Aging Male (2020) 23(5):607–18. doi: 10.1080/13685538.2018.1557139 30651030

[4] Wittert G Bracken K Robledo KP Grossmann M Yeap BB Handelsman DJ. Testosterone Treatment to Prevent or Revert Type 2 Diabetes in Men Enrolled in a Lifestyle Programme (T4DM): A Randomised, Double-Blind, Placebo-Controlled, 2-Year, Phase 3b Trial. Lancet Diabetes Endocrinol (2021) 9(1):32–45. doi: 10.1016/S2213-8587(20)30367-3 33338415

[5] Rallidis LS Kotakos C Tsalavoutas S Katsimardos A Drosatos A Rallidi M. Low Serum Free Testosterone Association With Cardiovascular Mortality in Men With Stable CAD. J Am Coll Cardiol (2018) 72(21):2674–5. doi: 10.1016/j.jacc.2018.08.2189 30466526

[6] Zhao SP Li XP. The Association of Low Plasma Testosterone Level With Coronary Artery Disease in Chinese Men. Int J Cardiol (1998) 63(2):161–4. doi: 10.1016/s0167-5273(97)00295-7 9510490

[7] Kloner RA Carson C 3rd Dobs A Kopecky S Mohler ER 3rd. Testosterone and Cardiovascular Disease. J Am Coll Cardiol (2016) 67(5):545–57. doi: 10.1016/j.jacc.2015.12.005 26846952

[8] Kim KH Jeong YT Oh H Kim SH Cho JM Kim YN. Autophagy Deficiency Leads to Protection From Obesity and Insulin Resistance by Inducing Fgf21 as a Mitokine. Nat Med (2013) 19(1):83–92. doi: 10.1038/nm.3014 23202295

[9] Salminen A Kauppinen A Kaarniranta K. FGF21 Activates AMPK Signaling: Impact on Metabolic Regulation and the Aging Process. J Mol Med (Berl) (2017) 95(2):123–31. doi: 10.1007/s00109-016-1477-1 27678528

[10] Yu D Ye X Wu Q Li S Yang Y He J. Insulin Sensitizes FGF21 in Glucose and Lipid Metabolisms *via* Activating Common AKT Pathway. Endocrine (2016) 52(3):527–40. doi: 10.1007/s12020-015-0801-9 26607153

[11] Chow WS Xu A Woo YC Tso AW Cheung SC Fong CH. Serum Fibroblast Growth Factor-21 Levels are Associated With Carotid Atherosclerosis Independent of Established Cardiovascular Risk Factors. Arterioscler Thromb Vasc Biol (2013) 33(10):2454–9. doi: 10.1161/ATVBAHA.113.301599 23887638

[12] Lee CH Woo YC Chow WS Cheung CYY Fong CHY Yuen MMA. Role of Circulating Fibroblast Growth Factor 21 Measurement in Primary Prevention of Coronary Heart Disease Among Chinese Patients With Type 2 Diabetes Mellitus. J Am Heart Assoc (2017) 6(6):e005344. doi: 10.1161/JAHA.116.005344 28588089 PMC5669163

[13] Richardson P McKenna W Bristow M Maisch B Mautner B O'Connell J. Report of the 1995 World Health Organization/International Society and Federation of Cardiology Task Force on the Definition and Classification of Cardiomyopathies. Circulation (1996) 93(5):841–2. doi: 10.1161/01.cir.93.5.841 8598070

[14] American Diabetes Association. 2. Classification and Diagnosis of Diabetes: Standards of Medical Care in Diabetes-2021. Diabetes Care (2021) 44(Suppl 1):S15–33. doi: 10.2337/dc21-S002 33298413

[15] Mancia G Fagard R Narkiewicz K Redán J Zanchetti A Böhm M. ESH/ESC Guidelines for the Management of Arterial Hypertension: The Task Force for the Management of Arterial Hypertension of the European Society of Hypertension (ESH) and of the European Society of Cardiology (ESC). Eur Heart J (2013) 34(28):2159–219. doi: 10.1093/eurheartj/eht151 23771844

[16] Levey AS Stevens LA Schmid CH Zhang YL Castro AF 3rd Feldman HI. A New Equation to Estimate Glomerular Filtration Rate. Ann Intern Med (2009) 150(9):604–12. doi: 10.7326/0003-4819-150-9-200905050-00006 PMC2763564 19414839

[17] Itoh N Ornitz DM. Evolution of the Fgf and Fgfr Gene Families. Trends Genet (2004) 20(11):563–9. doi: 10.1016/j.tig.2004.08.007 15475116

[18] Lewis JE Ebling FJP Samms RJ Tsintzas K. Going Back to the Biology of FGF21: New Insights. Trends Endocrinol Metab (2019) 30(8):491–504. doi: 10.1016/j.tem.2019.05.007 31248786

[19] Xiaolong L Dongmin G Liu M Zuo W Huijun H Qiufen T. FGF21 Induces Autophagy-Mediated Cholesterol Efflux to Inhibit Atherogenesis *via* RACK1 Up-Regulation. J Cell Mol Med (2020) 24(9):4992–5006. doi: 10.1111/jcmm.15118 PMC7205825 32227589

[20] Geng L Lam KSL Xu A. The Therapeutic Potential of FGF21 in Metabolic Diseases: From Bench to Clinic. Nat Rev Endocrinol (2020) 16(11):654–67. doi: 10.1038/s41574-020-0386-0 32764725

[21] Fisher FM Maratos-Flier E. Understanding the Physiology of FGF21. Annu Rev Physiol (2016) 78:223–41. doi: 10.1146/annurev-physiol-021115-105339 26654352

[22] Adua E Roberts P Sakyi SA Yeboah FA Dompreh A Frimpong K. Profiling of Cardio-Metabolic Risk Factors and Medication Utilisation Among Type II Diabetes Patients in Ghana: A Prospective Cohort Study. Clin Transl Med (2017) 6(1):32. doi: 10.1186/s40169-017-0162-5 28879491 PMC5587509

[23] Zhang Y Yan J Yang N Qian Z Nie H Yang Z. High-Level Serum Fibroblast Growth Factor 21 Concentration is Closely Associated With an Increased Risk of Cardiovascular Diseases: A Systematic Review and Meta-Analysis. Front Cardiovasc Med (2021) 8:705273. doi: 10.3389/fcvm.2021.705273 34513950 PMC8427036

[24] Shen Y Ma X Zhou J Pan X Hao Y Zhou M. Additive Relationship Between Serum Fibroblast Growth Factor 21 Level and Coronary Artery Disease. Cardiovasc Diabetol (2013) 12:124. doi: 10.1186/1475-2840-12-124 23981342 PMC3766150

[25] Dai Q Fan X Meng X Sun S Su Y Ling X. FGF21 Promotes Ischaemic Angiogenesis and Endothelial Progenitor Cells Function Under Diabetic Conditions in an AMPK/NAD+-Dependent Manner. J Cell Mol Med (2021) 25(6):3091–102. doi: 10.1111/jcmm.16369 PMC7957202 33599110

[26] Zeng Z Zheng Q Chen J Tan X Li Q Ding L. FGF21 Mitigates Atherosclerosis *via* Inhibition of NLRP3 Inflammasome-Mediated Vascular Endothelial Cells Pyroptosis. Exp Cell Res (2020) 393(2):112108. doi: 10.1016/j.yexcr.2020.112108 32445748

[27] Lin Z Pan X Wu F Ye D Zhang Y Wang Y. Fibroblast Growth Factor 21 Prevents Atherosclerosis by Suppression of Hepatic Sterol Regulatory Element-Binding Protein-2 and Induction of Adiponectin in Mice. Circulation (2015) 131(21):1861–71. doi: 10.1161/CIRCULATIONAHA.115.015308 PMC4444420 25794851

[28] Hui X Feng T Liu Q Gao Y Xu A. The FGF21-Adiponectin Axis in Controlling Energy and Vascular Homeostasis. J Mol Cell Biol (2016) 8(2):110–9. doi: 10.1093/jmcb/mjw013 26993043

[29] Lorigo M Mariana M Lemos MC Cairrao E. Vascular Mechanisms of Testosterone: The non-Genomic Point of View. J Steroid Biochem Mol Biol (2020) 196:105496. doi: 10.1016/j.jsbmb.2019.105496 31655180

[30] Rovira-Llopis S Bañuls C de Marañon AM Diaz-Morales N Jover A Garzon S. Low Testosterone Levels are Related to Oxidative Stress, Mitochondrial Dysfunction and Altered Subclinical Atherosclerotic Markers in Type 2 Diabetic Male Patients. Free Radic Biol Med (2017) 108:155–62. doi: 10.1016/j.freeradbiomed.2017.03.029 28359952

[31] Hotta Y Kataoka T Kimura K. Testosterone Deficiency and Endothelial Dysfunction: Nitric Oxide, Asymmetric Dimethylarginine, and Endothelial Progenitor Cells. Sex Med Rev (2019) 7(4):661–8. doi: 10.1016/j.sxmr.2019.02.005 30987932

[32] Troncoso MF Pavez M Wilson C Lagos D Duran J Ramos S. Testosterone Activates Glucose Metabolism Through AMPK and Androgen Signaling in Cardiomyocyte Hypertrophy. Biol Res (2021) 54(1):3. doi: 10.1186/s40659-021-00328-4 33546773 PMC7863443

[33] Dias JP Shardell MD Carlson OD Melvin D Caturegli G Ferrucci L. Testosterone vs. Aromatase Inhibitor in Older Men With Low Testosterone: Effects on Cardiometabolic Parameters. Andrology (2017) 5(1):31–40. doi: 10.1111/andr.12284 27792869 PMC5794008

[34] Kalicińska E Wojtas K Majda J Zacharski M Skiba J Śliwowski J. Expression of Sex Steroid Receptors and Aromatase in Adipose Tissue in Different Body Regions in Men With Coronary Artery Disease With and Without Ischemic Systolic Heart Failure. Aging Male (2020) 23(2):141–53. doi: 10.1080/13685538.2018.1494144 30193537

[35] Gagliano-Jucá T Basaria S. Testosterone Replacement Therapy and Cardiovascular Risk. Nat Rev Cardiol (2019) 16(9):555–74. doi: 10.1038/s41569-019-0211-4 31123340

[36] Rao PM Kelly DM Jones TH. Testosterone and Insulin Resistance in the Metabolic Syndrome and T2DM in Men. Nat Rev Endocrinol (2013) 9(8):479–93. doi: 10.1038/nrendo.2013.122 23797822

